# A closer look into maternal psychological distress and the associated factors: the case of autism and chronic conditions

**DOI:** 10.3389/fpsyt.2025.1461040

**Published:** 2025-05-23

**Authors:** Alexandra Cobzeanu, Ioan-Alex Merlici, Daria Elena Ionescu, Iulia Cristina Roca

**Affiliations:** ^1^ Department of Education Sciences, Faculty of Psychology and Education Sciences, Alexandru Ioan Cuza University, Iaşi, Romania; ^2^ Department of Surgery II, Faculty of Medicine, Grigore T. Popa University of Medicine and Pharmacy, Iaşi, Romania

**Keywords:** mothers, distress, children, autism, chronic disease

## Abstract

**Introduction:**

Mothers caring for children with autism or chronic illnesses may experience heightened psychological distress due to the ongoing demands associated with caregiving. This study aimed to examine levels of psychological distress among Romanian mothers of children with autism, chronic illness, or typical development, and to explore the role of health-related quality of life and emotion regulation strategies.

**Methods:**

A total of 211 Romanian mothers aged 20 to 67 years (M = 38, SD = 8.07) participated in this study. The sample included mothers of children with autism (30.8%), chronic illness (29.4%), and typical development (39.8%). Participants completed self-report questionnaires assessing psychological distress, emotion regulation strategies (cognitive reappraisal and expressive suppression), and health-related quality of life.

**Results:**

Health-related quality of life was found to be a significant predictor of psychological distress across all participant groups. Additionally, mothers of children with autism reported significantly higher levels of psychological distress compared to mothers of typically developing children.

**Discussion:**

The findings underscore the psychological burden experienced by mothers of children with autism and highlight the importance of targeted support interventions. Addressing psychological distress and its contributing factors may improve the well-being of these caregivers. The results carry important implications for mental health practitioners, researchers, and policymakers aiming to support families managing childhood disability or chronic illness.

## Introduction

Parenting stress represents a universal phenomenon, experienced by all parents, in varying degrees, although its prevalence is often accentuated by several factors such as challenging life situations, poverty, divorce, or single parenthood (1). In this context, parents of children with physical or mental conditions experience a significant amount of stress, often dealing with more parenting stress than parents of typically developed children (2). Previous research also suggests that parents of children with autism spectrum disorder (ASD) might be particularly vulnerable to experiencing higher levels of parental stress, even when compared to parents of children with other neurodevelopmental disorders or chronic conditions (3).

Previous research suggested that mothers of children with special needs might present higher levels of psychological distress when compared to the fathers (4). Moreover, the current research literature suggests that mothers’ parental stress might have a stronger impact on children’s behavior problems when compared to the parental stress of fathers (5). These differences might be explained by several factors, including (but not limited to) pregnancy, giving birth, and spending more time with the infants (6). However, it is important to note that this pattern might be consistent even several years after the birth of the child, with mothers presenting higher levels of distress. Moreover, while mothers’ stress might be more influenced by social isolation, spouse relationship problems, and perceived incompetence, fathers’ stress might be better explained by social isolation and health problems (7). While both mothers and fathers might have a strong impact on the cognitive and social development of their children, mothers’ parenting might have a stronger impact on curbing the development of behavior problems among children (5). The between maternal well-being and the challenges posed by raising children with specific needs, raised by diagnoses such as ASD or chronic conditions, has become a subject of increasing importance within the realm of psychological research (8). The need to explore maternal psychological distress in the context of raising children with ASD and chronic conditions stems from the significant impact that this may have on both mothers and the overall family dynamics (9–11), as highlighted by previous studies (12, 13).

In the current study, we aimed to investigate psychological distress among Romanian mothers, and the focus on this specific population was motivated by several factors. First, Romania offers a unique cultural framework to examine the proposed variables, given its ongoing transition from communism – a culture of segregation, to an inclusive society (14). Second, while there is a growing body of research addressing maternal psychological distress in the context of raising children with an autism spectrum disorder (ASD) and chronic diseases, studies that focus on specific Eastern European cultural contexts, such as Romania, are relatively scarce (15). Further, Romanian parents of children with special needs might be more likely to report a lower quality of life and less likely to expect the state to provide help when compared to their counterparts from other nations, both in Europe and in other regions (16).

The Double ABCX framework (17) describes how individuals cope and adapt to stressful conditions throughout time. In the context of parental experiences related to having a child with ASD, this model offers a conceptual framework for understanding the factors underlying parental distress and how families react to such stressors (15). The factors described by the model refer to (aA) child characteristics (e.g., the diagnosis and the associated behaviors, service needs, financial issues), (bB) internal and external resources (e.g., self-esteem, emotion regulation, perceived social support), (cC) appraisal of stressors – i.e., perceptions (e.g., psychological well-being, quality of life), and (BC) coping strategies and the way (xX) families adapt. According to the available literature exploring this model, the xX factor can be understood as either an “end result” or a “sequence of events”, which can be a favorable outcome (when families successfully modify their roles, routines, and other interactions to meet the new demands associated with the child’s diagnosis), or an unfavorable response (maladaptation) to the aA factor, resulting in a crisis (18).

**Figure 1:**
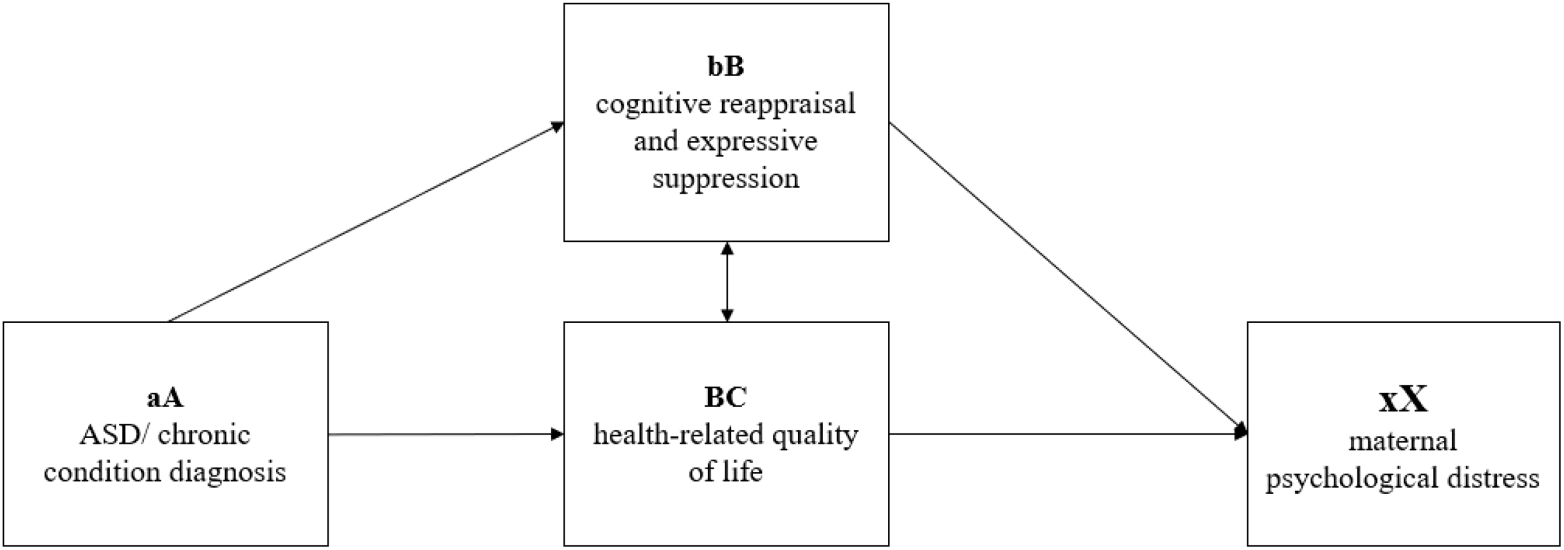
The proposed research framework, based on an adopted version of the modified Double ABCX model (17).

In the present study, we focused on ASD and chronic conditions as reflecting the aA factor in the Double ABCX framework, two emotion regulation strategies (i.e., cognitive reappraisal and expressive suppression) as an expression of the bB factor (internal resources), health-related quality of life as the cC factor (appraisal of stressors), and maternal psychological distress as the xX factor – which was the primary focus of this study (see **Figure 1**).

### Maternal psychological distress

The current research literature suggests that parents of children with ASD generally report higher levels of psychological distress and a lower quality of life compared to the parents of typically developed (TD) children (19). Also, mothers of children with special needs might present higher levels of psychological distress when compared to the fathers (4), and maternal distress might have a stronger impact on children’s behaviors compared to the parental stress of fathers (5). A recent review suggested that single young mothers of children with ASD who employ maladaptive coping strategies, exhibiting problem behaviors and sensory issues, are more susceptible to experiencing elevated levels of parental stress and poorer mental health (20).

Further, mothers of children diagnosed with a chronic condition (e.g., inflammatory bowel diseases, asthma, diabetes, cystic fibrosis, celiac disease) often report elevated levels of psychological distress due to the complexity of medical regimes (21), financial demands, and the complex management of unpredictable situations related to their medical condition (22). They also often experience guilt and worries, not only related to their child’s medical condition but also related to their other siblings, with a negative impact on the family system as a whole (23). The significantly higher psychological distress is generally higher than that of caregivers of healthy children, as systematic reviews previously highlighted (24). However, to date, no studies explored the specific roles of cognitive reappraisal, expressive suppression), and health-related quality of life among Romanian mothers of children with ASD, chronic conditions, and TD children, as in the present study.

### Emotion regulation strategies

Emotion regulation represents a set of cognitive skills aimed at managing emotions, in order to minimize the harmful impact of strong emotions. (25). The process of emotion regulation occurs in a concurrent manner with the emotions that the individual is feeling, individuals being determined to present strong emotional reactions toward significant events, then, through emotion regulation, they return to a state without strong emotions (26). Developing appropriate emotion regulation skills is important both for the mother’s well-being and for the proper development of the emotional skills of their children (27). Parents with better emotion-regulation skills often exhibit more positive parenting behavior and generally have children with better emotion regulation skills (28). The adoption of maladaptive emotion regulation strategies might increase parental stress (29), which further predict lower levels of parents’ bonding with their children.

In this study, we focused on two emotion regulation strategies, i.e., cognitive reappraisal and expressive suppression, as conceptualized by Gross and John (30). Cognitive reappraisal refers to regulating emotions by altering an individual’s interpretation or perception of a circumstance to adjust its emotional effect. For instance, when confronted with a difficult scenario, one may attempt to reframe the event in a more optimistic manner, emphasizing potential benefits or different viewpoints (31). Cognitive reappraisal was previously suggested as a more effective strategy for managing parental stress, parents that adopt this strategy are less likely to report parental burnout and more likely to manage negative emotions even when receiving insufficient social support (32). While the previous study focused on parents of typical children, similar effects were reported for parents of children with mental conditions, cognitive reappraisal being suggested as the most beneficial emotion regulation strategy (33). Moreover, the endorsement of cognitive reappraisal is also associated with better parental caregiving for children diagnosed with ASD (34). Furthermore, in the case of parents of children with a chronic condition or ASD, instead of solely viewing the diagnosis as a source of distress, parents employing cognitive reappraisal might think that this experience may strengthen family bonds as they support each other through this journey, may connect with other parents sharing this experience, or may gain a deeper understanding of their child’s needs. Thus, cognitive reappraisal can entail a deliberate alteration of their interpretation of this situation, fostering a more optimistic (and less stressful) perspective (35).

On the other hand, expressive suppression involves inhibiting the outward expression of one’s emotions, essentially masking or hiding one’s true feelings. For instance, a parent of a child with a chronic condition might inhibit the outward expression of their emotions to maintain composure and support their child. They might suppress their sadness, avoid showing tears, and maintaining a neutral or positive facial expression (36). Though this strategy may lead to short-term emotional regulation, it can negatively affect interpersonal relationships and long-term well-being due to the inhibition of genuine emotional expression (37).

As the available literature suggests, generally, cognitive reappraisal – compared to expressive suppression, contributes to a better psychological adjustment in various challenging situations among parents of children with ASD and chronic conditions (38). Effective emotion regulation strategies such as cognitive reappraisal may enhance the quality of parent-child interactions while decreasing parental psychological distress (34, 35). However, less effective strategies such as expressive suppression seem to have the opposite effect, contributing to increased levels of parental psychological distress (37, 39).

### Health-related quality of life

Health-Related Quality of Life (HRQoL) is a multidimensional concept referring to an individual’s perception of their health and the numerous ways in which it affects them in their daily lives. In addition to conventional indicators of health, such as the absence of disease or illness, it encompasses subjective assessments of one’s mental, physical, and social welfare (40).

Parents of children with disabilities or chronic illnesses are particularly susceptible in terms of the impact on their quality of life about health (41). The available literature suggests that parents of children with ASD or chronic conditions often report lower HRQoL, higher psychological distress, and lower overall quality of life compared to parents of TD children (42–44) and that mothers’ HRQoL in these cases seems to be lower than fathers’ (45, 46). Moreover, mothers of children with ASD are more likely to report lower HRQoL and present a higher likelihood of dealing with mental disorders (47). Generally, parents of children with ASD report a lower HRQoL, which is also associated with higher levels of parental stress (48). More recent meta-analytical reports also suggest that parents of children with mental disorders are experiencing a clinically relevant reduction in their quality of life, compared to the parents of typical children (49). Some scholars also reported that parents of children with ASD also reported lower HRQoL compared to caregivers of children with chronic conditions (50).

Nevertheless, there seems to be a bidirectional relation between parental psychological distress and HRQoL, which is highlighted by several studies. For instance, many scholars suggested that lower well-being and higher parental stress among parents of children with ASD seem to significantly predict lower HRQoL (51). At the same time, low HRQoL was suggested as a significant associated factor (52) and a significant predictor of psychological distress (53), though this direction is less investigated; thus, we aimed to add to the related literature by focusing on the predictive role of HRQoL on maternal psychological distress.

### The present study

The current study was built on the Double ABCX framework, focusing on two emotion regulation strategies (i.e., cognitive reappraisal and expressive suppression) and HRQoL as potential predictors of psychological distress among mothers of children with ASD, a chronic condition, or TD children.

Based on previous studies, the primary hypotheses of this study were the following:

*H1.* Cognitive reappraisal would be negatively associated with maternal psychological distress, regardless of the child’s status (with ASD, a chronic condition, or TD).

*H2.* Expressive suppression would be negatively associated with maternal psychological distress, regardless of the child’s status (with ASD, a chronic condition, or TD).

*H3.* HRQoL would be positively associated with maternal psychological distress, regardless of the child’s status (with ASD, a chronic condition, or TD).

*H4.* Cognitive reappraisal would negatively predict maternal psychological distress, regardless of the child’s status (with ASD, a chronic condition, or TD).

*H5.* Expressive suppression would positively predict maternal psychological distress, regardless of the child’s status (with ASD, a chronic condition, or TD).

*H6.* HRQoL would positively predict maternal psychological distress, regardless of the child’s status (with ASD, a chronic condition, or TD).

*H7*. Maternal psychological distress would be significantly higher among mothers of children with ASD and chronic conditions, compared to mothers of TD children, with the highest rates among mothers of children with ASD.

## Materials and methods

### Participants and procedure

The sample comprised 211 Romanian mothers aged 20 to 67 (M = 38, SD = 8.07). Most had two children (46.4%) and were married (85.3%). Among the participants, 30.8% (N = 65) reported having a child with ASD, 29.4% (N = 62) reported having a child with a chronic disease, and 39.8% (N = 84) reported they had TD children (see **Table 1** for a detailed description). The chronic diseases reported by the participants included epilepsy, ADHD, Rett syndrome, KBG syndrome, and tetraparesis.

**Table 1:** Sample descriptives (N = 211).

Variable		
Number of children	N	%
1	84	39.8
2	98	46.4
3	21	10
>3	8	3.8
Children	N	%
* ASD*	65	30.8
* chronic disease*	62	29.4
* typical development*	84	39.8

A web-based questionnaire was distributed using social media groups (i.e., Facebook and Whatsapp); also, personal e-mail invitations were distributed at the end of 2022 and the beginning of 2023 (12.11.2022- 19.03.2023). Participants were briefed about the participation prerequisites, incentives, and their prerogative to exit the study at will. Furthermore, we assured all participants that their input would remain confidential and unidentified, solely intended for this research. The research protocol was designed following the ethical guidelines from the university where the authors are affiliated and the 2013 Helsinki Declaration. Following their informed consent, participants needed around 10 minutes to answer all the questions.

### Measures

#### Psychological distress

We measured participants’ psychological distress using the DASS-21 scale developed by Lovibond and Lovibond (54). In the present study, we used the Romanian version of the scale (14). The 21 items measured participants’ depression, anxiety, and stress symptoms, which were self-reported considering the preceding week. Participants gave their answers on a Likert scale ranging from 0 (did not apply at all) to 3 (very applicable). Example items included “I couldn’t seem to experience any positive feeling at all” (depression); “I was aware of dryness of my mouth” (anxiety); and “I found it hard to wind down” (stress). Higher scores indicated higher psychological distress. Cronbach’s alpha in the present study was.95.

#### Emotion regulation

We used the 10-item Emotional Regulation Questionnaire developed by Gross and John (30) to measure two emotion regulation strategies, i.e., cognitive reappraisal (6 items) and expressive suppression (4 items). The Romanian version that we used was previously used in studies among Romanian samples (55) and showed good psychometric properties. Example items include “I keep my emotions to myself” (expressive suppression), and “When I want to feel more positive emotion (such as joy or amusement), I change what I’m thinking about” (cognitive reappraisal). Items were measured on a 7-point Likert scale, where 1 = strong disagreement, and 7 = strong agreement. The internal consistency (Cronbach’s alpha-s) was α=.86 for the cognitive reappraisal subscale, and α=.80 and for expressive suppression. Higher scores indicated a higher tendency to use one of the two strategies.

#### Health-related quality of life

We used the Health-Related Quality of Life (WB-HRQoL) scale developed by Janković et al. (40), which is a 19-item instrument measuring physical (5 items), mental health (8 items), social (three items), and environmental (three items) quality of life. Example items included “I do not feel any pain” (physical aspects of quality of life), “I am always in a good mood” (mental health aspects), “My family relations are excellent”, and “I regularly meet my friends and enjoy their company” (social aspects). Participants responded using a 5-point Likert scale ranging from 1 (I do not agree completely”) to 5 (I agree completely). Cronbach’s alpha-s for each dimension were the following: 0.70 for the physical dimension, 0.81 for mental health, 0.71 for the social dimension, and 0.77 for the environmental dimension. The instrument was translated from the English language into the Romanian language using the forward-backward translation design (56), and the few discrepancies between the original and the back-translated version were identified and solved, resulting in the final versions of the scale. Cronbach’s alpha for the overall scale (measuring global health-related quality of life) was 0.89. Higher scores indicated higher health-related quality of life.

A demographic scale assessed participants’ age, number of children, and child’s status (i.e., with ASD, a chronic disease, or no diagnosis – which will further be referred to as TD children).

## Results

### Overview of data analysis

We used the 26v. of the SPSS program to analyze our data. There were no missing data, as the items were set as required (i.e., mandatory) to complete the study. The assumptions were first evaluated using descriptive univariate analysis (see **Table 2**). Skewness and Kurtosis indicators were calculated to determine the distributions’ normality (57).

**Table 2:** Descriptive statistics for the main variables (N = 211).

Variable	M	SD	Min	Max	Skewness	Kurtosis
Overall sample						
**Psychological distress**	22.98	14.58	0	63	.41	-.39
**Cognitive reappraisal**	29.45	7.91	6	42	-.49	-.14
**Expressive suppression**	14.14	6.10	4	29	.39	-.47
**Health-related quality of life**	59.40	13.18	19	89	-.26	.32
Mothers – typical dev. children						
**Psychological distress**	19.57	13.61	0	54	.40	-.81
**Cognitive reappraisal**	28.92	7.82	6	42	-.77	.58
**Expressive suppression**	13.36	6.16	4	29	.41	-.38
**Health-related quality of life**	62.14	11.39	34	89	-.01	-.14
Mothers – children with ASD						
**Psychological distress**	25.27	14.57	0	61	.28	-.41
**Cognitive reappraisal**	29.61	8.18	12	42	-.27	-.74
**Expressive suppression**	14.41	5.83	4	28	.17	-.79
**Health-related quality of life**	57.07	14.41	19	86	-.46	.14
Mothers – children with chronic diseases						
**Psychological distress**	25.20	15.18	0	63	.48	-.09
**Cognitive reappraisal**	30	7.85	11	42	-.42	-.40
**Expressive suppression**	14.91	6.28	4	29	.59	-.35
**Health-related quality of life**	58.14	13.65	23	89	.06	.59

### Associations between the main variables

Next, we examined the associations between the primary variables in the overall sample, as well as separately – depending on the targeted groups (i.e., mothers of TD children/children with ASD/children with a chronic disease diagnosis - CD). Results are presented in **Tables 3A**-**D**.

**Table 3a:** Correlations between the main variables (overall sample, N = 211).

Variable	1	2	3	4
1. Psychological distress	–			
2. Cognitive reappraisal	-.04	–		
3. Expressive suppression	.32**	.21*	–	
4. HRQoL	-.57**	.15*	-.22**	–
5. Age	-.17*	.03	-.06	.09

*p <.05; **p <.001; QoL, Quality of life.

**Table 3b:** Correlations between the main variables (TD children, N = 84).

Variable	1	2	3	4
1. Psychological distress	–			
2. Cognitive reappraisal	.02	–		
3. Expressive suppression	.45**	.18	–	
4. HRQoL	-.46**	.19	-.21	–
5. Age	-.22*	.15	.05	.18

*p <.05; **p <.001; HRQoL, Health-related Quality of Life.

**Table 3c:** Correlations between the main variables – mothers of children diagnosed with ASD, N = 65.

Variable	1	2	3	4
1. Psychological distress	–			
2. Cognitive reappraisal	-.08	–		
3. Expressive suppression	.28*	.28*	–	
4. HRQoL	-.73**	.20	-.32*	–
5. Age	-.10	-.10	-.16	.09

*p <.05; **p <.001; HRQoL, Health-related Quality of Life **Table 3d**.

**Table 3d:** Correlations between the main variables – mothers of children diagnosed with a chronic condition, N = 62.

Variable	1	2	3	4
1. Psychological distress	–			
2. Cognitive reappraisal	-.12	–		
3. Expressive suppression	.17	.16	–	
4. HRQoL	-.46**	.09	-.11	–
5. Age	-.17	-.03	-.18	-.02

**p <.001; HRQoL, Health-related Quality of Life.

#### Overall sample

Results suggested that psychological distress was negatively associated with HRQoL, *r* = -.57, *p* <.001 and age, *r* = -.17, *p* = .01. Also, psychological distress was positively associated with expressive suppression, *r* = .32, *p* <.001 (**Table 3A**).

#### Parents of TD children

Results suggested that psychological distress was negatively associated with HRQoL, *r* = -.46, *p* <.001 and age, *r* = -.22, *p* = .03. Also, psychological distress was positively associated with expressive suppression, *r* = .45, *p* <.001 (**Table 3B**).

#### Parents of children with ASD

Results suggested that psychological distress was negatively associated with HRQoL, *r* = -.73, *p* <.001 and positively associated with expressive suppression, *r* = .28, *p* = .02 (**Table 3C**).

#### Parents of children with a chronic condition

Results suggested that psychological distress was negatively associated with HRQoL, *r* = -.46, *p* <.001. No other significant associations emerged (**Table 3D**).

### Hierarchical regression analysis predicting maternal psychological distress (depending on child’s status)

Next, we conducted a hierarchical regression analysis to predict maternal psychological distress. We performed the analyses in the overall sample and separately (depending on the child’s diagnosis). In all cases, we introduced age in Step 1, expressive suppression in Step 2, and health-related quality of life in Step 3 (see **Tables 4A**–**D**).

**Table 4a:** Hierarchical regression results – (overall sample, N = 211).

Model	Variables	B(SE)	β	R^2^	ΔR^2^
Model 1				.02	.02
	Age	-.30 (.12)	-.17*		
Model 2				.12	.11
	Age	-.26 (.11)	-.14*		
	Expressive suppression	.75 (.15)	.31**		
Model 3				.37	.36
	Age	-.19 (.10)	-.11*		
	Expressive suppression	.47 (.13)	.20**		
	HRQoL	-.57 (.06)	-.51**		

*p <.05; **p <.001; HRQoL, Health-related Quality of Life.

**Table 4b:** Hierarchical regression results – (mothers – TD children, N = 84).

Model	Variables	B(SE)	β	R^2^	ΔR^2^
**Model 1**				.05	.04
	Age	-.34 (.16)	-.22*		
**Model 2**				.26	.24
	Age	-.38 (.14)	-.25*		
	Expressive suppression	1.02 (.21)	.46**		
**Model 3**				.37	.35
	Age	-.28 (.13)	-.18*		
	Expressive suppression	.85 (.20)	.38**		
	Health-QoL	-.41 (.11)	-.34**		

*p <.05; **p <.001; HRQoL, Health-related Quality of Life.

**Table 4c:** Hierarchical regression results – (mothers – children with ASD, N = 65).

Model	Variables	B(SE)	β	R^2^	ΔR^2^
**Model 1**				.01	-.004
	Age	-.21 (.25)	-.10		
**Model 2**				.08	.05
	Age	-.12 (.25)	-.06		
	Expressive suppression	.69 (.30)	.27*		
**Model 3**				.54	.52
	Age	-.06 (.17)	-.03		
	Expressive suppression	.12 (.23)	.04		
	Health-QoL	-.72 (.09)	-.71**		

*p <.05; **p <.001; HRQoL, Health-related Quality of Life.

**Table 4d:** Hierarchical regression results – (mothers - children with a chronic disease, N=62).

Model	Variables	B(SE)	β	R^2^	ΔR^2^
**Model 1**				.02	.01
	Age	-.33 (.24)	-.17		
**Model 2**				.05	.01
	Age	-.27 (.25)	-.14		
	Expressive suppression	.35 (.31)	.14		
**Model 3**				.25	.21
	Age	-.32 (.22)	-.16		
	Expressive suppression	.21 (.28)	.08		
	Health-QoL	-.50 (.12)	-.45**		

**p <.001; HRQoL, Health-related Quality of Life.

#### Overall sample

The first model (**Table 4A**), which only included age, was significant, *F*(1, 210) = 6,19, *p* = .01, explaining 2.4% in the variance of psychological distress. The second model, which added expressive suppression, was significant, *F*(2, 210) = 15.17, *p* <.001, and explained 11.90% of the variance in psychological distress. The change brought by the addition of this variable was also significant, *Fch* = 23.48, *p* <.001. Model 3, which added HRQoL, was also significant, *F*(3, 210) = 41,85, *p* <.001; the change brought by the addition of this variable was also significant, *Fch* = 83.20, *p* <.001. The final model explained 36.9% of the variance in mothers’ psychological distress. In the final model, all predictors were significant, but the strongest was HRQoL, *β* = -.51, *p* <.001, followed by expressive suppression, *β* = .20, *p* <.001, and age, *β* = -.11, *p* = .04.

#### Parents of TD children

The first model (**Table 4B**), which only included age, was significant, *F*(1, 83) = 4.42, *p* = .03 explaining 4% in the variance of psychological distress. The second model, which added expressive suppression, was significant, *F*(2, 83) = 14.71, *p* <.001, and explained 24.80% of the variance in psychological distress. The change brought by the addition of this variable was also significant, *Fch* = 23.77, *p* <.001. Model 3, which added HRQoL, was also significant, *F*(3, 83) = 16.23, *p* <.001; the change brought by the addition of this variable was also significant, Fch = 14.41, *p* <.001. The final model explained 35.5% of the variance in mothers’ psychological distress. In the final model, all predictors were significant, but the strongest was expressive suppression, *β* = .38, *p* <.001, followed by HRQoL, *β* = -.34, *p* <.001, and age, *β* = -.18, *p* = .04.

#### Parents – children with ASD

The first model (**Table 4C**), which only included age, was not significant, *F*(1, 64) = .72, *p* = .39. The second model, which added expressive suppression, was not significant, *F*(2, 64) = 2.90, *p* =.06; the change brought by the addition of this variable was, however, significant, *Fch* = 5.04, *p* =.02. Model 3, which added HRQoL, was the only significant model, *F*(3, 64) = 24.56, *p*<.001; the change brought by the addition of this variable was significant, *Fch* = 62.14, *p*<.001. The final model explained 52.5% of the variance in mothers’ psychological distress. In the final model, the only significant predictor was HRQoL, *β* = -.71, *p*<.001.

#### Parents – children with a chronic disease

The first model (**Table 4D**), which only included age, was not significant, *F*(1, 61) = 1.78, *p* = .18. The second model, which added expressive suppression, was also not significant, *F*(2, 61) = 1.54, *p*=.22. Model 3, which added HRQoL, was the only significant model, *F*(3, 61) = 6.63, *p*<.001; the change brought by the addition of this variable was significant, *Fch* = 62.14, *p*=.001. The final model explained 52.5% of the variance in maternal psychological distress. In the final model, the only significant predictor was HRQoL, *β* = -.71, *p*<.001.

Finally, to better understand the potential differences in mothers’ psychological distress (depending on their child’s status/condition), we performed One-way ANOVA analyses. Results suggested significant differences between the groups, *F* (2, 210) = 3.93*, p*=.02. More specifically, the mothers of children diagnosed with ASD (*M*=25.27) reported marginally significantly higher psychological distress (*Mdif*=-5.70, *p*=.052) than those with TD (*M*=25.20). No other significant differences emerged.

## Discussions

Through the current study, we aimed to investigate psychological distress experienced by mothers of TD children, children with ASD, and children with chronic illnesses, through the lens of the Double ABCX framework (17). For the aA factor of the framework, child characteristics, we focused on investigating children with TD, ASD, and chronic illnesses. We operationalized the bB factor (i.e., internal and external resources) through two emotion regulation strategies, cognitive reappraisal, and expressive suppression, respectively. The cC factor (i.e., appraisal of stressors) was operationalized through HRQoL, and, finally, the xX factor was operationalized through maternal psychological distress. Moreover, we included the age of our participants as a potential predictor of maternal psychological distress.

In the overall sample, HRQoL was the strongest predictor of maternal psychological distress, with higher levels of HRQoL being associated with lower levels of distress. The second strongest predictor was expressive suppression, having a positive effect on maternal psychological distress, followed by age, which had a negative effect. Therefore, the cC factor (i.e., appraisal of stressors) represented the most important predictor of psychological distress among our participants. These results are in line with previous research, with parents of children with disabilities or physical or psychological disorders presenting lower levels of HRQoL, compared to parents of TD children (41).

In the group of mothers of TD children, expressive suppression was the strongest predictor of psychological distress, having a positive effect, followed by HRQoL and age, both having a negative impact. For this group, the bB factor represented the most important predictor of maternal psychological distress. The current results suggest that expressive suppression might be a more relevant predictor of parental psychological distress for mothers of TD children when compared to cognitive reappraisal. The endorsement of maladaptive emotion regulation strategies might be more impactful toward psychological distress, compared to the adoption of adaptive strategies.

When investigating the group of mothers of children with ASD, HRQoL was the only significant predictor of maternal psychological distress, with higher levels of HRQoL being associated with lower levels of distress. The current results are in line with the previous literature, which suggests that parents of children with ASD usually report lower levels of HRQoL when compared to other parental groups (48). Furthermore, our results suggest that Romanian parents of children with ASD are particularly vulnerable to reduced levels of HRQoL, this factor being more important for predicting their levels of psychological distress.

Finally, in the group of mothers of children with chronic diseases, HRQoL was the only significant predictor, having a negative effect on maternal psychological distress. The final model for the group in this case followed a similar pattern to the model for the group of mothers of children with ASD. The current results suggest that parents of children with special needs might follow a similar pattern in developing psychological distress, with HRQoL playing a particularly relevant role in the Romanian context. Nevertheless, further research is needed to test the validity of these claims.

An important finding that diverged from our expectations was represented by the absence of a significant effect of cognitive reappraisal toward maternal psychological distress in all the investigated groups. These results seem counterintuitive, considering previous research that suggests that cognitive reappraisal might be a more efficient mechanism for reducing psychological distress, compared to expressive suppression (32). However, the current results might suggest that HRQoL might be particularly relevant in predicting psychological distress for Romanian mothers of children with special needs, to the point where emotion regulation strategies might no longer make a significant difference. Finally, when comparing the three groups, mothers of children with ASD presented higher levels of psychological distress. These results are convergent with previous research (3), which suggests that parents of children diagnosed with ASD are experiencing higher levels of psychological distress compared to other parents.

### Limitations and future directions

Several limitations of the current study need to be addressed. First, we collected our data through an online survey, which limited our ability to control potential confounding variables that might have influenced the responses offered by our participants. To counter this limitation, future studies should consider using a more controlled environment (i.e., a laboratory) for data collection. Second, we operationalized our research variables through questionnaires, which relied on subjective evaluations reported by our participants. Furthermore, using questionnaires increased the risk of desirability bias in the responses offered by our participants. To counter this limitation, future studies should employ experimental designs allowing more objective measurements of the research variables. Third, we used a convenience sample, which does not reflect the characteristics of the investigated demographic group. To counter this limitation, future studies might benefit from using representative samples that better reflect the characteristics of the investigated population.

Fourth, through the current study, we focused on a limited number of variables related to maternal psychological distress. However, we did not consider several variables that might be relevant in predicting maternal psychological distress. For example, social support was previously suggested to play a crucial role in predicting psychological distress among parents (58). Moreover, parental psychological distress might further be accentuated by poverty, divorce, and single parenthood (1). Therefore, future studies might benefit from introducing various variables that contribute to explaining psychological distress.

Finally, in the current study, we focused on investigating mothers of TD children, children with ASD, and children with chronic diseases. Various disorders and medical conditions experienced by children might have varying effects on the mental well-being of their parents. Moreover, we did not compare the parents whose children presented different chronic illnesses. Furthermore, future research might benefit from investigating parents of children with other diagnoses (e.g., Down syndrome) (59).

### Implications

The current study provides relevant data on the Romanian cultural context of mothers of children with special needs. Our findings provide various practical implications for practitioners, researchers, and policymakers. First, for the groups of mothers of children with ASD and mothers of children with chronic diseases, HRQoL was the only significant predictor of psychological distress. The cultural context might play an important role in the observed results, with Romanian parents of children with special needs being particularly vulnerable to presenting a lower HRQoL, while also being less likely to expect support from the authorities (16). Therefore, policymakers should consider interventions that aim to address this problem by providing more social, material, and emotional support for parents of children with special needs. Addressing these needs might prove more beneficial than investing in interventions aimed toward the development of emotion regulation skills because neither expressive suppression nor cognitive reappraisal was a significant predictor of maternal psychological distress in the two groups of parents of children with special needs. However, further research is needed to test the validity of these claims.

Second, cognitive reappraisal had no significant effect on psychological distress in any of the models. In contrast, expressive suppression had a negative effect on psychological distress in the overall sample and the group of mothers of children with TD. These results seem unexpected, considering previous research suggested that cognitive reappraisal might be one of the most adaptive emotion regulation strategies (33). However, these findings suggest that interventions aimed at developing emotion regulation skills for Romanian parents might benefit from diminishing the negative impact of maladaptive coping strategies rather than enhancing cognitive reappraisal. Nevertheless, more research is needed, to explore the relevance of various emotion regulation strategies.

Third, age had a negative impact on psychological distress for mothers of children with TD. However, age was no longer a significant predictor of psychological distress for mothers of children with special needs. These results suggest that for mothers of TD children, psychological distress might become easier to manage with age. However, it is important to note that, generally speaking, mothers of higher ages might also have children of higher ages, which might also explain the decrease in psychological distress. Nevertheless, for mothers of children with special needs, age no longer played a significant role. This might suggest that raising children with ASD or chronic disorders might represent a significant source of psychological distress, regardless of their age. Therefore, interventions aimed at diminishing parental psychological distress might be efficient for various age groups.

## Conclusion

The current study contributed to the current research literature by investigating the predictive factors of parental psychological distress in Romania. HRQoL was the most relevant predictor, followed by expressive suppression and age. Expressive suppression was a stronger predictor for psychological distress among mothers of TD children. In comparison, HRQoL was a stronger predictor for psychological distress among mothers of children with ASD or chronic diseases. The current study brought valuable insights into the Romanian context of parental psychological distress, highlighting a potentially stronger impact of HRQoL on parental distress, compared to emotion regulation strategies, and age. In the Double ABCX framework, appraisal of stressors represented the most important factor in predicting maternal psychological distress, followed by internal resources. Moreover, in our study, we observed a similar pattern to the one previously reported in the current research literature, suggesting that mothers of children with ASD experience higher levels of psychological distress, compared to mothers of TD children and children with other conditions.

## Data Availability

The raw data supporting the conclusions of this article will be made available by the authors, without undue reservation.
